# OpenNavSense platform: A low-cost, open-source inertial navigation system for the evaluation of estimation algorithms

**DOI:** 10.1016/j.ohx.2024.e00621

**Published:** 2024-12-31

**Authors:** Pablo Raul Yanyachi, Jorch Mendoza-Chok, Brayan Espinoza-Garcia, Juan Carlos Cutipa Luque, Daniel Yanyachi Aco Cardenas

**Affiliations:** Instituto de Investigacion Astronomico y Aeroespacial Pedro Paulet, Universidad Nacional de San Agustin de Arequipa, 04000, Arequipa, Peru

**Keywords:** Development platform, FreeRTOS, Global positioning system, Inertial navigation system, Low-cost, Micro-electromechanical, Open-source

## Abstract

Inertial navigation systems (INS) are widely used in commercial aviation, maritime navigation, and unmanned vehicle guidance. However, these systems are often sensitive, costly, and challenging to access. To address these limitations, an open-source, low-cost platform named INS OpenNavSense has been developed. This platform is built using FreeRTOS, an open-source real-time operating system (RTOS) that enables the microcontroller to run parallel individual threads (tasks), providing a practical and effective tool for implementing estimation algorithms that compensate for the use of low-cost microelectromechanical systems (MEMS) sensors instead of high-end sensors in professional INS. The main contribution of this work is the introduction of a FreeRTOS-based platform, which facilitates independent management of computational and processing tasks. The platform incorporates accelerometers, gyroscopes, magnetometers, Global Positioning System (GPS) module, and barometer sensors. Sensor data is calibrated and filtered to enhance accuracy, offering researchers a robust and reliable tool for testing their estimation algorithms. To validate this platform, the open-source Mahony library was used for attitude and heading reference system estimation, demonstrating the types of algorithms that can be tested. Tests were conducted with a drone carrying the platform as payload, and results from this low-cost INS were compared to the drone’s INS, showing both similarity and viability as a development platform.

**Table:** 

**Specifications table**	

Hardware name	INS OpenNavSense by IAAPP
Subject area	Educational tools and open-source alternatives to existing infrastructure

Hardware type	•Measuring physical properties and in-lab sensors • Field measurements and sensors

Closest commercial analog	VectorNav VN-300
Open source license	GNU General Public License (GPL) 3.0
Cost of hardware	50 $
Source file repository	https://osf.io/a9jg2

## 1 Hardware in context

Inertial navigation systems (INS) are essential instruments in manned and unmanned vehicles, as they provide real-time information about vehicle dynamics to the onboard computer or operator. These systems accurately deliver data such as orientation, velocity, acceleration, position, and altitude [1], [2]. INS are increasingly being integrated into new devices, including electric vehicles, drones, autonomous underwater vehicles (AUVs), unmanned surface vehicles (USVs), remotely operated vehicles (ROVs), and even modern household appliances such as smart vacuum cleaners.

Based on the precision and stability of the microelectromechanical systems (MEMS) in the inertial measurement units (IMUs), which typically include accelerometers and gyroscopes, INSs are classified into five categories: marine-grade, military-grade, aviation-grade, tactical-grade, and consumer-grade. The costs of each vary widely depending on the application and required precision. The top categories are marine-grade (≥$1M) and military-grade (≥$100K) INSs. The intermediate category comprises aviation-grade INSs (≥$20K), primarily used in commercial air navigation. Tactical-grade INSs (≥$2K) are used in weapons guidance and unmanned aerial vehicles (UAVs). Finally, consumer-grade INSs, which start at (≥$10), are commonly used in consumer electronics [3], [4]. Among the most well-known consumer-grade INSs are the (MPU-6050, InvenSense, USA), (LSM9DS1, STMicroelectronics, Switzerland), and (BMI160, Bosch Sensortec, Germany).

One of the most well-known tactical-grade INS on the market is the (VN-300, VectorNav Technologies, USA). This system employs advanced sensor fusion techniques, error correction, self-calibration, and dual Global Navigation Satellite System (GNSS) antennas to provide precise position and orientation relative to true north. Priced at approximately $5000, the (VN-300, VectorNav Technologies, USA) is widely used in research prototypes, USVs and UAVs. However, this cost is prohibitively high for projects with limited funding.

Therefore, several studies and initiatives have attempted to achieve similar results using low-cost, consumer-grade accelerometers, gyroscopes, magnetometers, Global Positioning System (GPS) modules, and barometers that often lack calibration and whose technical specifications are not well documented [5], [6], [7]. These studies employ Kalman filter-based algorithms for sensor fusion, signal filtering, and processing, as well as GPS-based correction algorithms, which often necessitate the creation of custom hardware tailored to each of them. For example, in [8], the authors aim to improve the accuracy of orientation estimation using adaptive Kalman filters, but the hardware used for testing is tailored to their specific application. Similarly, [6] employs Kalman-based sensor fusion algorithms to estimate attitude angles, while [7] applies an Attitude and Heading Reference System (AHRS) algorithm based on Euler angles, utilizing the (LSM6DSOX, STMicroelectronics, Switzerland) and comparing its performance with the (VN-300, VectorNav Technologies, USA) achieving comparable results [9]. As the reader can observe, each of these studies develops its specific hardware, inspiring further advancements in this field by combining the strengths of each approach.

Besides, a limited number of open-source projects are available for testing navigation algorithms, with most options focusing on simulation software rather than hardware. Examples of open-source software include the AutoNAV project [10], which simulates the use of odometry and range sensors in GNSS-denied environments, and the system described by [11], which incorporates both simulation and real-world post-processed data to validate its algorithms. Regarding hardware, an important initiative is the OpenIMU project developed by [12], which offers an open-source 9 Degrees of Freedom (DOF) sensor designed for real-time performance. However, only open-access software is available, and it lacks open-access hardware, meaning the main components and designs are not publicly available.

Given the aforementioned problem, this work presents the open-source, low-cost INS platform **INS OpenNavSense**, which serves as a useful tool for implementing estimation algorithms within a real-time open-source operating system by executing tasks in parallel. This platform builds upon the previous work of [7], which analyzed the background noise and stability of the (LSM6DSOX, STMicroelectronics, Switzerland) accelerometer and gyroscope. One of its key innovations lies in the coordinated execution of tasks in parallel and the independent execution of threads within each task. Furthermore, the architecture provides flexibility and adaptability for implementing modern Kalman-based estimation algorithms for INS, making it suitable for academic research. For validation, the platform incorporates the Mahony AHRS algorithm described in [13], with a detailed explanation in Appendix A, and the obtained results are compared and evaluated against navigation data from a commercial drone equipped with tactical-grade sensors.

## 2 Hardware description

The platform consists of electronic components, including a microcontroller, sensor modules, and a GPS module. To select the appropriate hardware for the **INS OpenNavSense**, several components were studied, as detailed in the following paragraphs.

Table B.1 presents the microcontrollers evaluated for the proposed prototype, including the (ESP32, Espressif Systems, China), the (ESP8266, Espressif Systems, China), the (Arduino Uno, Arduino.cc, Italy), and the (Raspberry Pi Zero, Raspberry Pi Foundation, UK). Although the (Arduino Uno, Arduino.cc, Italy) has lower power consumption, it lacks FreeRTOS capability and native wireless connectivity, requiring the use of external modules. The (ESP8266, Espressif Systems, China) was not selected due to its moderate computational performance and limited FreeRTOS compatibility, narrowing the choice between the (ESP32, Espressif Systems, China) or (Raspberry Pi Zero, Raspberry Pi Foundation, UK). After comparing these two controllers, the (ESP32, Espressif Systems, China) was chosen for the prototype due to its well-known computational capabilities, integration of Bluetooth BLE, low power consumption, native support for real-time operating systems, and lower cost compared to the (Raspberry Pi Zero, Raspberry Pi Foundation, UK).

For the IMU, Table B.2 summarizes the IMUs studied for the proposed prototype. The options include the (MPU6050, InvenSense, USA), the (MPU9250, InvenSense, USA), the (LSM6DSOX, STMicroelectronics, Switzerland), and the (BMI160, Bosch, Germany). Although the (LSM6DSOX, STMicroelectronics, Switzerland) is the most expensive among them, it has the lowest Root Mean Square (RMS) noise in both the accelerometer and the gyroscope. These values were previously analyzed and studied in [7], demonstrating excellent stability comparable to (VN-300, VectorNav Technologies, USA) MEMS. For GPS positioning, Table B.3 summarizes the proposed GPS modules. The options include the (BN880, Beitian, China), the (NEO-6M, u-blox, Switzerland), (NEO-M8M, u-blox, Switzerland), and (ZED-F9P, u-blox, Switzerland). The (BN880, Beitian, China) module was selected due to its 10Hz refresh rate, active receiving antenna, and incorporation of the (HMC5883L, Honeywell, USA) magnetometer, which is useful for correcting and estimating orientation. A similar analysis is performed for the barometer, as shown in Table B.4, which presents the evaluated candidates. The options include the (BMP180, Bosch, Germany), (BMP280, Bosch, Germany), (BME280, Bosch, Germany), and the (LPS22HB, STMicroelectronics, Switzerland). The (BMP180, Bosch, Germany) chip was selected to measure pressure and estimate altitude primarily due to its availability in the local market. However, it can be replaced or upgraded to a sensor with better characteristics if needed.

With the selected components, the **INS OpenNavSense** platform provides 10-DOF functionality (three-axis accelerometer, three-axis gyroscope, three-axis magnetometer, and a barometer) and offers GPS positioning. A microSD card module is employed for data logging, while the Bluetooth BLE protocol of the (ESP32, Espressif Systems, China) is used for working mode selection and real-time transmission of navigation data. Additonally, the **INS OpenNavSense** was designed as an open-source platform that includes the design files for Printed Circuit Boards (PCBs) and 3D printing. The current version discussed in this article was constructed using Polylactic Acid (PLA) filament, a biodegradable material, for 3D printing, along with the assembly of the boards through PCB fabrication services. Its adaptable design offers the necessary versatility for mounting on various vehicles, including drones, UAVs, cars, bicycles, and laboratory prototypes. Furthermore, it employs two 18 650 lithium-ion batteries, guaranteeing a sustained operational duration of at least 12 hours.

One of the main advantages of this platform is its use of the FreeRTOS operating system, which allows for the execution of tasks in parallel according to their priorities, ensuring the independent operation of each task [14], [15]. By working with FreeRTOS, it is possible to implement estimation algorithms of any kind, making it an ideal option for training, research, and fast prototyping. Additionally, the platform features working modes for sensor calibration, used in conjunction with user interfaces that estimate magnetometer bias coefficients as well as gyroscope and accelerometer offsets. These features enable the acquisition of more accurate and reliable measurements, with a maximum update rate of 20 Hz. Finally, it is important to mention that the proposed hardware for this platform is economical, with an approximate cost of $50, and offers potential applications such as:

• Recording raw parameters such as linear acceleration, angular velocity, and magnetic field during missions or testing.
• Serving as a platform for the real-time implementation of sensor calibration algorithms.
• Acting as a backup INS for manned and unmanned vehicles.

## 3 Design files summary

### 3.1 Hardware files

• Top Case Piece: It is the upper part of the platform, in .stl format, designed for 3D printing. This piece is essential for holding the internal components and features a 6 mm diameter hole in the center for attaching the payload to the drone.
• Bottom Case Piece: It is the bottom part of the platform, also in .stl format, designed for 3D printing. This piece supports the internal components and has cylindrical parts for securing the electronic board with bolts.
• Board Design: The printed electronic circuit connects all the onboard sensors. It is attached with bolts to the Bottom Case Piece.

**Table 1:** List of design files with open source code license.

Design filename	File type	Open source license	Location of the file
Top Case Piece	3D model (*.stl)	GPL3	https://osf.io/j7gve
Bottom Case Piece	3D model (*.stl)	GPL3	https://osf.io/ftq3a
Board Design	Kicad (*.pcb)	GPL3	https://osf.io/wjpq6

### 3.2 Software files

• Main code: This is the main program that runs FreeRTOS. It reads the onboard sensors, manages task execution, collects data, performs calculations, and outputs information about the parameters of the entire platform.
• Functions: This is the support file where the functions used in the main code are declared.
• Schematic Design: This is the design file for the electronic connections of the sensors, regulator, battery, GPS, and microcontroller.

**Table 2:** List of design files with open source code license.

Design filename	File type	Open source license	Location of the file
Main code	Arduino (*.ino)	GPL3	https://osf.io/ug43f
Functions	Arduino (*.ino)	GPL3	https://osf.io/4qfp3
Schematic Design	Kicad (*.sch)	GPL3	https://osf.io/epqba

## 4 Bill of materials summary

See Table 3.

**Table 3:** List of components for the project.

Designator	Component	Number	Cost per unit - currency	Total cost - currency	Source of materials	Material type	DataSheet
Micro controller	ESP WROOM 32	1	US $ 4	US $ 4	https://cutt.ly/9367Iyn	Other	https://bit.ly/3Czf5V5

GPS Module	BN880	1	US $ 13	US $ 13	https://cutt.ly/M367KNK	Other	https://bit.ly/3O1FKfQ

IMU	LSM6DSOX	1	US $ 17	US $ 17	https://cutt.ly/43674Kc	Other	https://bit.ly/3CntC6p

Barometer	BMP180	1	US $ 1	US $ 1	https://cutt.ly/Q365eAf	Other	https://bit.ly/40LBRDl

Battery	18650 Litio	2	US $ 2.5	US $ 5.00	https://cutt.ly/p365or6	Other	https://bit.ly/3VaS5T9

Voltage Regulator	L7805 TO-220	1	US $ 1	US $ 1	https://cutt.ly/3365lgy	Other	https://bit.ly/3YN9DFw

Switch	Double Switch MTS 202	1	US $ 2.5	US $ 2.5	https://cutt.ly/b365Rm9	Other	https://bit.ly/4hM0TZ2

Capacitors	Capacitor polarized 10 μF	1	US $ 0.6	US $ 0.6	https://cutt.ly/S365UH8	Other	–

Screw	Stainless Steel Allen Hexagon Bolt, M3 x 16mm	8	US $0.25	US $2.00	https://cutt.ly/z367wUv	Other	–

## 5 Build instructions

### 5.1 Electronic circuit assembly

**Fig. 1:**
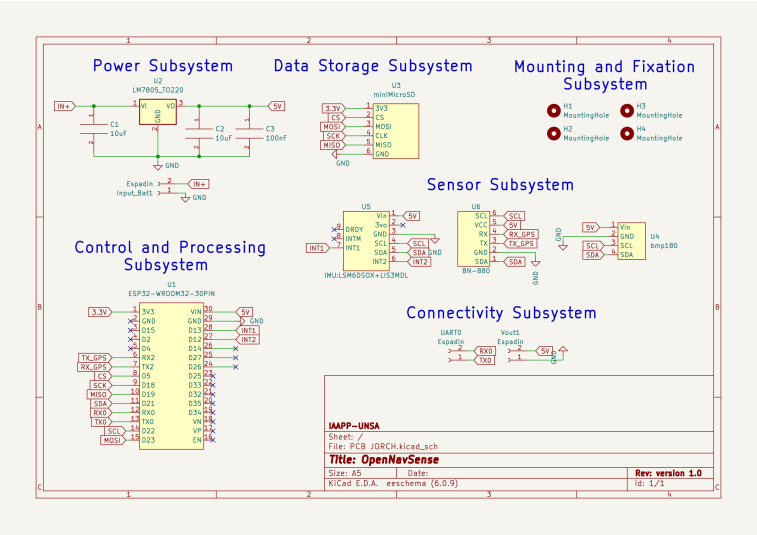
Schematic diagram of the electronic subsystems of the OpenNavSense platform.

The electronic diagram was developed using the KiCad 6.0.8 program. Fig. 1 shows the electronic connections of all component pins in the system, with each pin labeled for a better understanding of the electrical distribution. The system is divided into six subsystems:

• **Power Subsystem**: This subsystem rectifies and ensures a 5 V DC output for the system. The (LM7805, Texas Instruments, USA) chip provides an average power of 1 W to all the components on the board.
• **Data Storage Subsystem**: This subsystem is responsible for recording all onboard parameters on a microSD card. It consists of a module that connects to the main board through the SPI protocol. The module supports memory capacities of up to 64 GB in FAT32 format. The data, primarily floats and integers, are stored in *.txt* format using ASCII encoding.
• **Control and Processing Subsystem**: Composed solely of the ESP32-WROOM32 board, it functions as a microcomputer with the FreeRTOS operating system. It is responsible for receiving sensor data, calculating, managing tasks and peripherals, sending, and storing data.
• **Sensor Subsystem**: This section includes the onboard sensors, which use I2C and UART protocols to communicate with the control and processing subsystem. In this version, only three sensors are connected: (LSM6DSOX, STMicroelectronics, Switzerland), (BN880, Beitian, China), and (BMP180, Bosch, Germany).
• **Connectivity Subsystem**: This subsystem is responsible for debugging and updating the main program, serving as an alternative to the USB cable. It is useful for monitoring power consumption, recording edge changes on a digital oscilloscope, and externally powering the entire system with batteries or regulated power supplies.
• **Mounting and Fixation Subsystem**: Although not a subsystem, it is a crucial component, as the electronics must be securely attached to the platform.

#### 5.1.1 System architecture

**Fig. 2:**
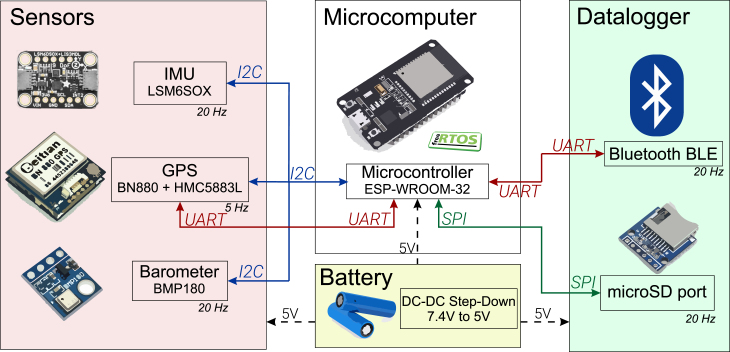
Architecture of INS OpenNavSense.

The system architecture is depicted in Fig. 2, illustrating the communication protocols employed by each sensor, along with their refresh rates and functions. On the left side, sensors responsible for recording environmental parameters communicate using the I2C protocol, while the GPS sensor utilizes UART exclusively. At the center of the architecture is the (ESP32, Espressif Systems, China) microcontroller, which handles computational tasks under the management of the FreeRTOS operating system. On the right side, a datalogger setup captures data from two devices: the microSD card stores all sensor data, while selected parameters from the code can be transmitted via Bluetooth. The system is powered by two batteries providing a total voltage of 7.4 V, regulated through a (LM7805, Texas Instruments, USA) linear regulator. Additionally, a 5 V power supply is implemented for all components to ensure greater compatibility with potential future sensors.

### 5.2 Software assembly instruction

**Fig. 3:**
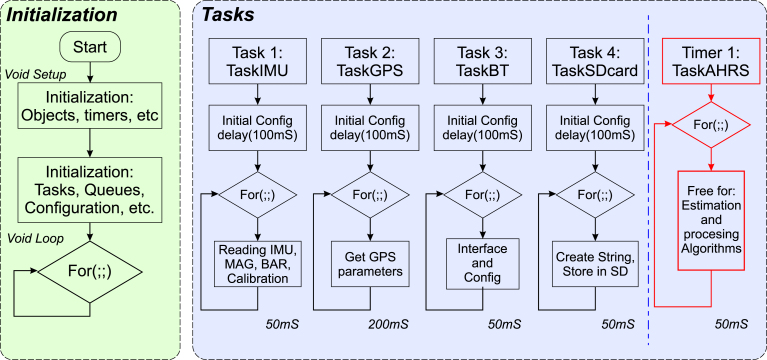
Flowchart of configuration and task execution in the control and processing subsystem.

In the ESP32-WROOM-32 module, the main program is located in the file “*main code.ino*”, as listed in Table 2. This subsection explains the internal operation of the program. Fig. 3 presents the execution flow diagram of the main program. The operation is similar to that of any Arduino script but differs in the configuration of library objects, microcontroller peripherals, task declarations, priorities, stack, and FreeRTOS timers, both before and during the execution of the ‘‘void setup’’ function. No code is added in the ‘‘void loop’’ function, as tasks are executed based on priority and within their assigned time.

On the left side of Fig. 3, the program initialization is depicted. It is important to note that adding, modifying, or removing tasks requires configuration during this stage. As the program runs on an (ESP32, Espressif Systems, China), which has sufficient core speed (80 MHz) and memory (512 kB) and is widely used for IoT applications [16], many tasks of considerable size can be created. However, it should be noted that this is not parallel computing, but sequential processing based on priorities [14]. On the right side, the defined tasks are illustrated. Four tasks and one timer have been set. The initial configuration is executed in each, followed by an infinite loop entry. This loop is configurable in terms of its duration, set to run at 20 Hz or 50 ms for all tasks and timers, except for the GPS. The timer task is emphasized, as it consistently adheres to the assigned period. It is possible to increase the operating frequency, but this would lead to higher energy consumption. The authors opted to maintain these refresh rates. Below is a detailed description of each task.

#### 5.2.1 TaskIMU

With an assigned memory space of 8192 bytes and a priority of 3, this task is responsible for collecting data from the accelerometer, gyroscope (LSM6DSOX, STMicroelectronics, Switzerland), magnetometer (HMC5883L, Honeywell, USA), and barometer (BMP180, Bosch, Germany). The task starts by configuring the operating speed and range of each sensor. Within the for loop, raw data from each sensor is collected, then calibrated, filtered, and stored in global variables. The execution of this task is critical for maintaining data quality, so any modifications should be carefully considered.

#### 5.2.2 TaskGPS

With an assigned memory space of 8192 bytes and a priority of 2, this task collects raw data from the (BN880, Beitian, China) GPS with GLONASS, including the number of satellites, latitude, longitude, altitude (in meters), speed, GPS time, and other parameters. The manufacturer specifies that this GPS module can operate at 10 Hz; however, it was decided to run it at 5 Hz, or a period of 200 ms. Since the module lacks a *Dataready* pin, synchronization, and prioritization are crucial to prevent data repetition or loss, as other navigation parameters can be derived from position readings.

#### 5.2.3 TaskBT

With an assigned memory space of 8192 bytes and a priority of 4, this task is responsible for configuring the module via Bluetooth through a simple user interface. In this task, the INS operation mode is defined, which can be one of the following: Magnetometer calibration mode, Accelerometer and gyroscope calibration mode, AHRS mode, or Off. This task has the lowest priority, as it primarily checks for the presence of a connected client and handles reading and sending strings of data.

#### 5.2.4 TaskSDcard

With an assigned memory space of 16 384 bytes and a priority of 1, this task records all variables in a text file. Based on the instructions from TaskBT, the appropriate “filepath” for recording the data is determined. By default, this task writes a 250-byte string containing 23 parameters to the SD memory every 50 ms.

#### 5.2.5 Timer1

This timer operates at a stable frequency of 20 Hz. It is software-based and functions independently of task priorities while maintaining the configured timing. This timer is dedicated to host estimation algorithms, as discrete-time systems require a constant execution period. By default, Mahony’s AHRS algorithm [13] is integrated with this timer.

Finally, Table 4 summarizes the reasons for each assigned priority, helping users to understand what can be modified to customize it according to their specific needs.

**Table 4:** Task priorities and reasons for selection.

Task	Priority	Reason for priority assignment
TaskSDcard	1	This task is assigned the highest priority as it handles data logging, ensuring that critical data is written to the SD card regularly and reliably. The default writing frequency (every 50 ms) requires a stable operation to prevent data loss.

TaskGPS	2	While GPS data is important for navigation, it operates at a lower frequency (5 Hz) compared to IMU data. The priority is set to 2 to allow sufficient processing time for other high-frequency tasks like TaskIMU while ensuring regular data collection from the GPS module.

TaskIMU	3	This task is assigned in three priority because it collects and processes data from the accelerometer, gyroscope, magnetometer, and barometer at a higher constant rate.

TaskBT	4	This task has the lowest priority because it primarily handles user interaction via Bluetooth, including calibration and mode selection. Since it deals with configuration and monitoring, it can tolerate delays without affecting system performance significantly.

Timer1	N/A	As a software-based timer operating at 20 Hz, it is not governed by task priorities but maintains consistent timing for estimation algorithms like Mahony’s AHRS, ensuring constant execution periods needed for accurate system performance.

### 5.3 Assembly instructions

**Fig. 4:**
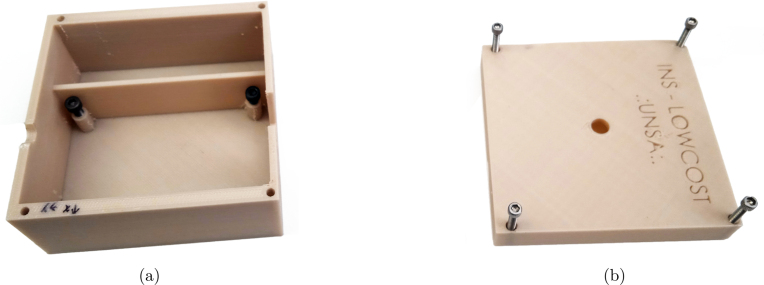
Views of the 3D printed model of the OpenNavSense INS platform. (a) Bottom Case Piece. (b) Top Case Piece.

To assemble the platform, the parts listed in Table 1 must first be 3D printed. These parts can be printed using any material; for this project, 1.75 mm PLA in beige color was used. Fig. 4 shows the two main parts of the platform, including the adjustable bolts of the plate and the junction of both parts. The platform was designed using SolidWorks 2023 software, and the original files are available in the project’s repository or at the following link:

• Bottom Case Piece: https://osf.io/gn7x9
• Top Case Piece: https://osf.io/3zu6y

**Fig. 5:**
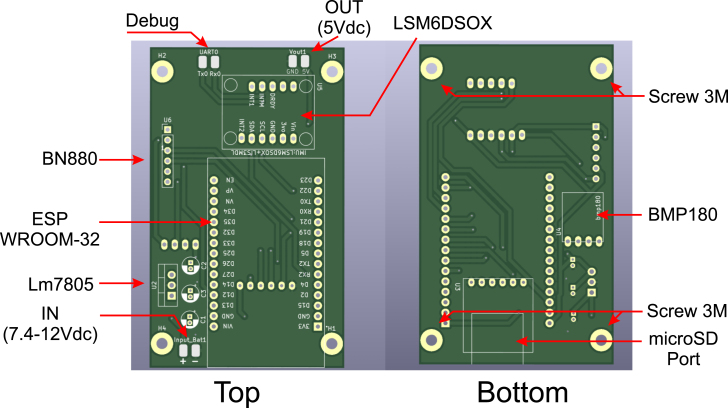
Circuit for PCB board printing, with component details.

The electronic board was manufactured using PCB fabrication services. The design file is provided in Kicad software format, along with the compressed Gerber file for manufacturing (https://osf.io/84wzp). Fig. 5 shows the components and their positions. Since it is a low-cost platform, it can also be fabricated on a prototyping board if necessary.

**Fig. 6:**
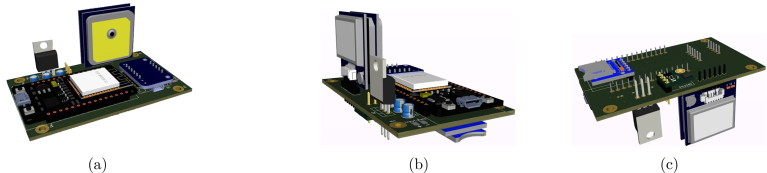
Views of the 3D model of the OpenNavSense INS platform. (a) Top right view, (b) Top left view, (c) Bottom view.

Once the components listed in Table 3 are soldered onto the electronic board, the result should resemble Fig. 6. The component layout in this design optimizes space utilization and facilitates access for removing or inserting the microSD card, as well as updating the (ESP32, Espressif Systems, China) script from the same side. Additionally, four holes are included for attachment with 3D-printed parts. The original design remains unchanged and can be found at the following link: https://osf.io/epqba.

**Fig. 7:**
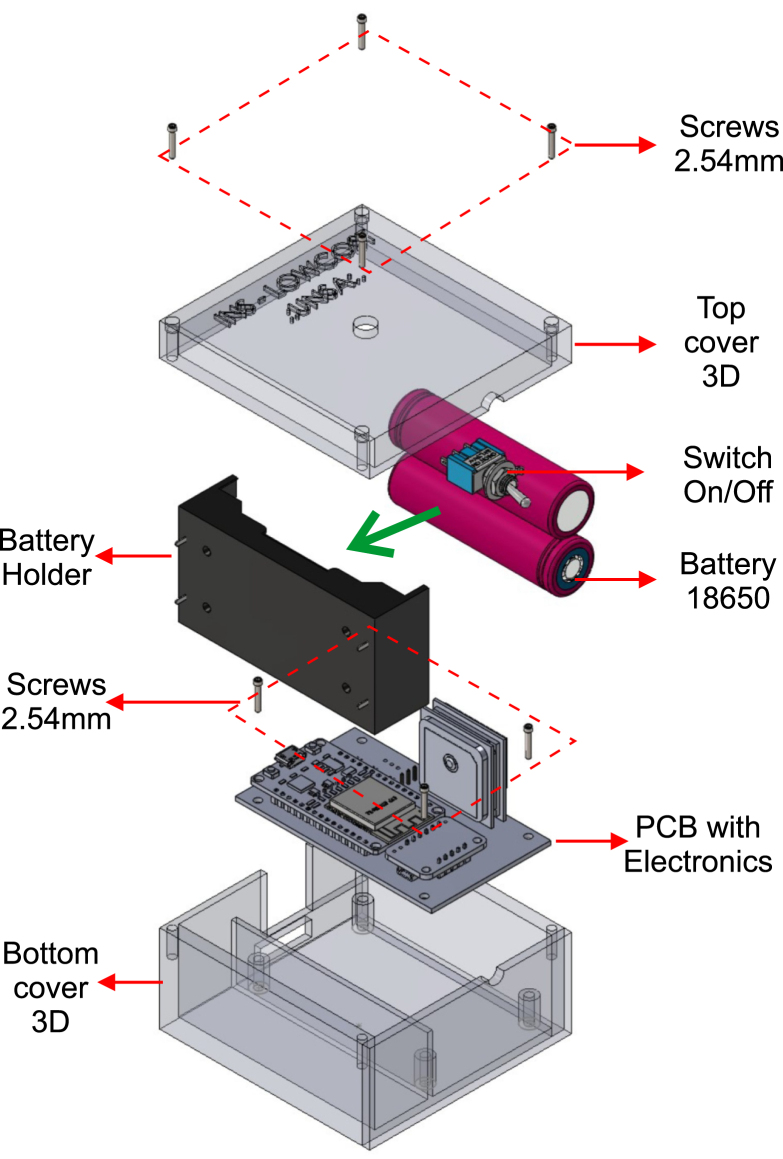
Exploded view of INS OpenNavSense with all components.

Finally, Fig. 7 illustrates all the internal components, ranging from the top bolts that secure the 3D upper part to the base, to the battery holder with two 18 650 batteries, the power on/off switch, the bolts that attach the electronic board to the base, the PCB with components, and the 3D base. With a total weight of 238.7 g and dimensions of 8.0 × 8.3 × 4.7 cm, this platform is a robust tool for the development and testing of position and orientation estimation algorithms.

## 6 Operation instructions

**Fig. 8:**
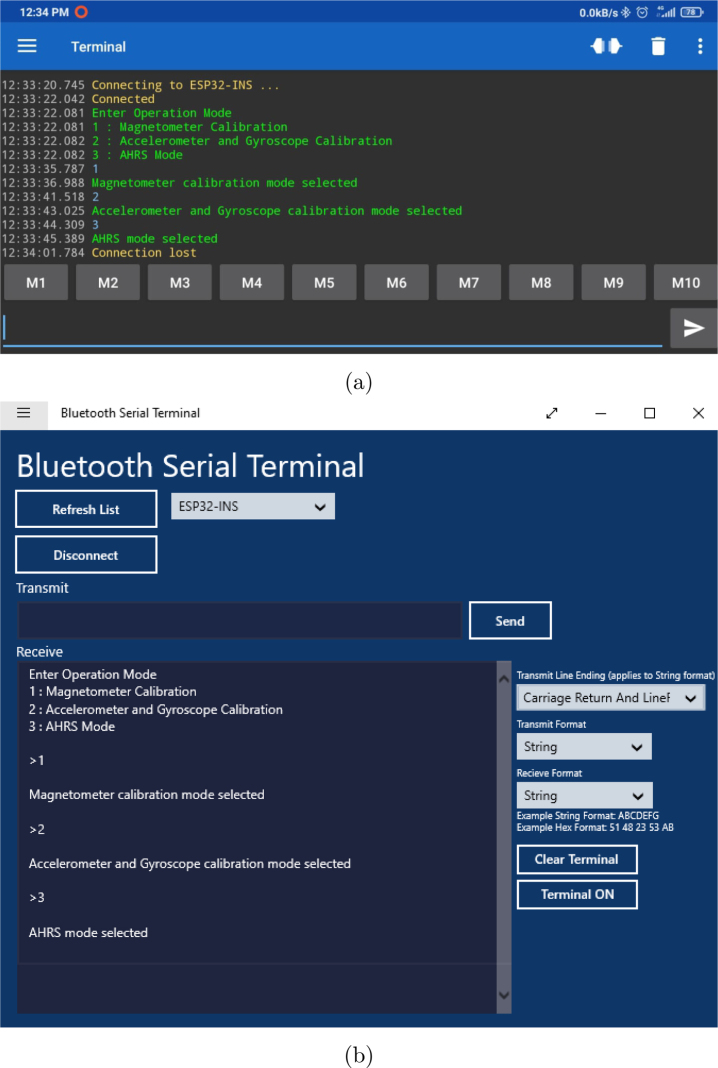
User interface via Bluetooth Serial Terminal on Android (a) and Windows (b).

The operation mode of the **OpenNavSense INS** platform is straightforward. A free application called “Serial Bluetooth Terminal” is used on Android to connect to the **OpenNavSense INS** Bluetooth named “ESP32-INS”. Once the Android device connects to the module, the user is prompted to select an operation mode. By default, the device begins saving raw data to a text file. Fig. 8(a) shows the available operation modes. However, the use of this device is not limited to Android; it is also compatible with Bluetooth Terminal applications on Windows (Fig. 8(b)), Linux, Mac, and other systems. One of the advantages of this connectivity is the ease of transferring raw data via Bluetooth to a computer, as shown in Fig. 8.

The operation modes include sensor calibration options such as “Magnetometer Calibration” and “Accelerometer and Gyroscope Calibration”. These modes must be performed before activating the “AHRS Mode”, which runs the INS algorithms to calculate the module’s orientation and position.

### 6.1 Calibration of magnetometer sensor

The calibration of the magnetometer involves recording the readings of the three axes in oscillatory movements in the working environment. The (HMC5883L, Honeywell, USA) sensor registers the minimum perturbations caused by nearby devices or materials that produce a magnetic field within a range of approximately 2 m. Without magnetometer calibration, the orientation estimation will be negatively affected. To ensure the accurate acquisition of true data from the Earth’s magnetic field, it is necessary to calculate the calibration matrices [17].

**Fig. 9:**
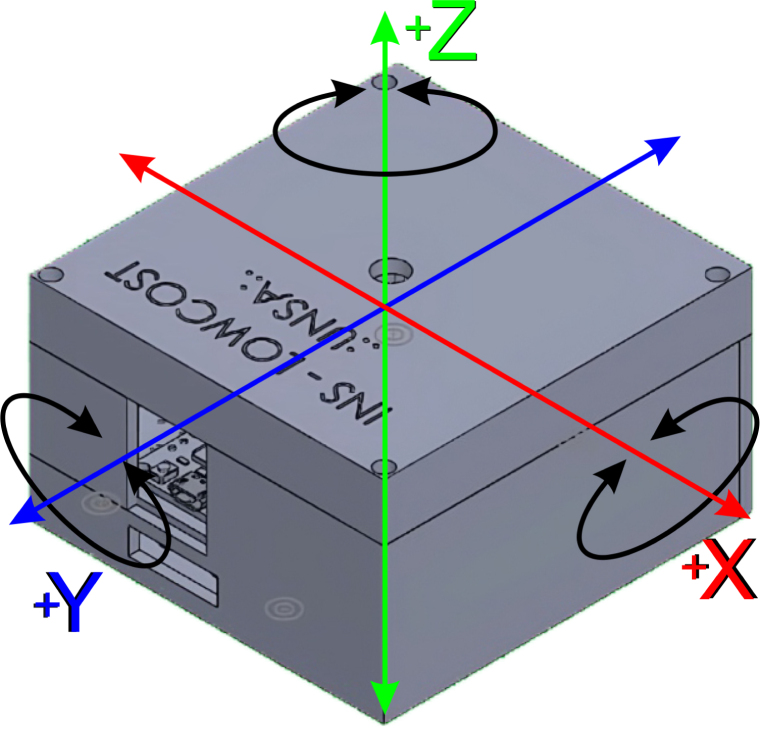
INS OpenNavSense with three-axis for rotation of magnetometer.

When mode 1 is selected, circular movements of 360 deg in the 3 axes must be initiated as shown in Fig. 9. The movements should be performed in all possible combined directions, that is, first rotational movements in the Z, Y, X axes, and then combining them in axes such as Z-Y, Y-X, and X-Z. The recommended duration for these maneuvers is at least 1 min.

### 6.2 Calibration of accelerometer and gyroscope sensor

When mode 2 is selected, data collection used to estimate the offset of the accelerometer and gyroscope will start. This task starts 5 s after selecting the option. In this mode, the module should be placed on a stable surface and there should be no disturbances or constant movement. This calibration is not affected by magnetic disturbances, so it can be performed in any stable location.

### 6.3 Update of calibration coefficients

After performing modes 1 and 2 of the **INS OpenNavSense** platform, the following files must be extracted from the micro SD memory:

• “/datalog_static.txt” : datalog of the static record, necessary to estimate the calibration coefficients of the accelerometer and gyroscope.
• “/datalog_dynamic.txt”: datalog of the dynamic record, necessary to estimate the Hard Iron and Soft Iron matrices of the magnetometer.

Once the files of the records of both modes are obtained, the calibration software that we provide for the inertial sensors on the INS **OpenNavSense** platform must be executed.

• “OpenNavSenseCalibration.exe” : https://osf.io/4urk6
• “Installer_mcr” : https://osf.io/p34ze

**Fig. 10:**
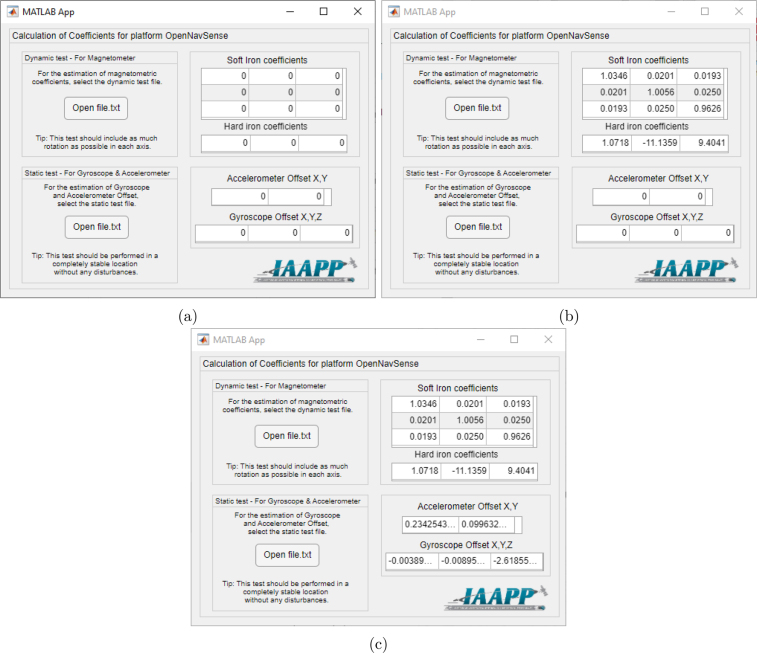
Calibration software for the INS OpenNavSense platform. (a) Initial screen, (b) Hard Iron and Soft Iron calculation, (c) Accelerometer and Gyroscope Offset calculation.

As a first step, you must install the software “Installer_mcr.exe”, which contains all the prerequisites to run the functions written in Matlab, without the need to have the complete program installed. Then you must run the program “OpenNavSenseCalibration.exe”. This program was developed with the Matlab App Designer tool to calculate the calibration values of the **INS OpenNavSense** in any environment in a simple way. When you run this program, the window shown in Fig. 10 will be displayed.

In Fig. 10(a), two buttons can be observed to select the files “/datalog_static.txt” and “/datalog_dynamic.txt”. The Soft Iron and Hard Iron coefficients corresponding to the magnetometer are shown at the top of the figure, while the offsets that must be calculated from the accelerometer and gyroscope are located at the bottom. Fig. 10(b) shows the estimated coefficients for the test environment used by the authors of this work, but these values will change significantly depending on the results of each user test. Due to the internal distribution of the platform components, there will always be different values than the identity matrix. The offsets of the accelerometer and gyroscope of the platform are shown in Fig. 10(c). All of new calculations must be updated in the main script (“Main code” in Table 2).

### 6.4 Instructions for using AHRS

After updating the calibration coefficients for the inertial sensors, the module is ready to be used in the mission to estimate orientation and position. These values are automatically saved in the internal memory and calculated according to the programmed estimation algorithm.

**Fig. 11:**
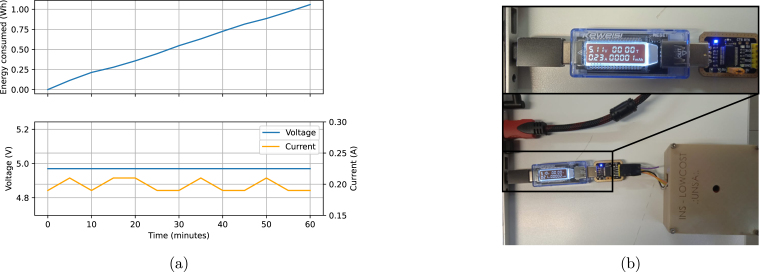
Energy consumption of the platform. (a) Energy consumption, voltage, and current graphs during 1 h of usage. (b) Photograph of energy consumption measurement using a USB meter and mAh consumption counter.

Regarding the autonomy of the platform, the consumption tests were carried out for an hour, and the energy consumption was estimated to be 1 Wh. With this value, the continuous operation of the system can be extended to a minimum of 12 h by using two 18 650 lithium-ion batteries. These batteries are widely used, and studies such as [18] further characterize them. Fig. 11(a) illustrates the energy usage, voltage, and current over one hour, while Fig. 11(b) portrays the platform during the measurement of energy consumption.

## 7 Validation and characterization

This section presents data collected from the **OpenNavSense** sensors, which are used to estimate the platform’s orientation, altitude, and location. The goal of this section is to characterize and demonstrate the platform’s viability as a tool for developing attitude and navigation estimation algorithms. Before conducting application tests with the DJI Phantom 4 Pro (P4P) drone, it is necessary to characterize and calibrate the proposed sensors.

### 7.1 Statistical analysis

**Fig. 12:**
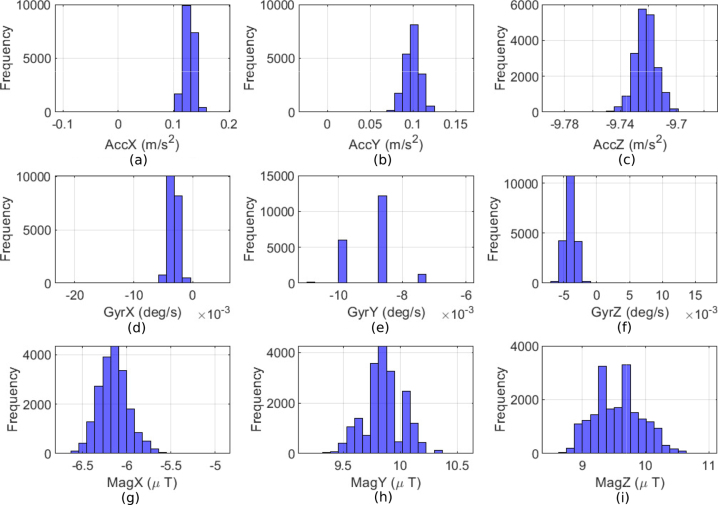
Statistical analysis of Raw IMU measurements. Accelerometer histogram in (a) X-axis (AccX), (b) Y-axis (AccY), (c) Z-axis (AccZ); Gyroscope histogram in (d) X-axis (GyrX), (e) Y-axis (GyrY), (f) Z-axis (GyrZ); and Magnetometer histogram in (g) X-axis (MagX), (h) Y-axis (MagY), and (i) Z-axis (MagZ).

A statistical analysis of the IMU is performed to characterize the proposed prototype. In Fig. 12, histograms are presented for each sensor axis over 1000 samples, captured during static measurements with no movement.

A preliminary histogram analysis reveals a positive skew in the accelerometer’s x-axis (AccX), indicating that most readings tend to have a positive offset. Similarly, the accelerometer’s y-axis (AccY) exhibits a rightward skew, although the data is more tightly clustered around the mean. In contrast, the accelerometer’s z-axis (AccZ) approximates a normal distribution, centered around −9.72 m/s${}^{2}$, corresponding to the gravitational force, which aligns with the Z-axis pointing upward.

Analyzing the gyroscope data, the distribution along the gyroscope x-axis (GyroX) is spread toward negative values. In the gyroscope y-axis (GyroY), the data is heavily skewed to the negative side, and the gyroscope z-axis (GyroZ) also shows a strong negative skew. Concerning the magnetometer data, the x-axis (MagX) appears to be normally distributed, centered around −6.1295 μT. The magnetometer data on the y-axis (MagY) is similarly approximately normally distributed but centered around 9.8572 μT. The magnetic field data on the z-axis (MagZ) shows a slightly broader spread around 9.5681 μT, indicating greater variability in the magnetic field measurements along the Z-axis. The distribution appears normal but with a wider range of values compared to MagX and MagY.

**Fig. 13:**
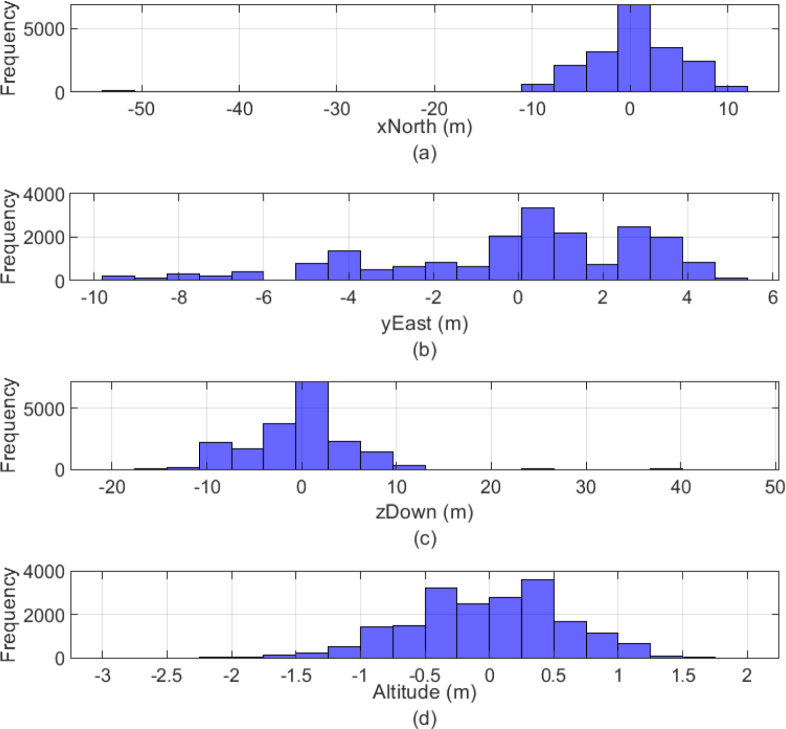
Statistical analysis of GPS and altitude calculated with the barometer measurements. GPS histogram of (a) North, (b) East, and (c) Down components. Barometer Histogram of (d) altitude.

To characterize the GPS module, the geodetic coordinates (latitude, longitude, and altitude) were transformed into North-East-Down (NED) coordinates relative to the reference point (−16.46561, −71.49306, 2.46795E3) using the MATLAB function geodetic2ned. Additionally, the altitude from the barometer data is calculated using (1), where $h_{\text{alt}}$ represents the altitude in (m) above sea level (m.a.s.l.), $P_{\text{sta}}$ is the measured pressure in (hPa), and $P_{0}$ is the standard sea level pressure in (hPa). The barometer altitude is calculated by subtracting the initial altitude in m.a.s.l. from the current altitude in m.a.s.l. With these calculations, the histogram of the measured data is shown in Fig. 13.

(1) $$h_{\text{alt}}=44330.0\cdot \left(1.0-{\left(\frac{P_{\text{sta}}}{P_{0}}\right)}^{0.1903}\right)$$

Fig. 13 shows that the GPS data exhibits variability across all axes, with the North (xNorth) component slightly skewed to the left, indicating fluctuations toward negative values (southward), while the East (yEast) component displays more symmetrical variation around zero but with a slight bias toward negative values (westward). The Down (zDown) component is heavily skewed to the right, suggesting significant fluctuations in altitude, with most values near zero but a long tail extending toward higher altitudes. In contrast, the barometer’s altitude data is more stable and symmetric around zero, indicating more consistent altitude measurements compared to the GPS data, particularly in the vertical axis. Overall, the GPS readings show more variability, especially in the vertical (zDown) direction, while the barometer provides more reliable altitude measurements.

Finally, Table 5 presents key statistical metrics for the raw measured data, including the mean, standard deviation, Root Mean Square (RMS), peak-to-peak value, and the population coefficient of skewness based on the third moment (skewness), which are described in Table C.5. Among those values, the skewness describes the symmetry of the data distribution, helping detect any bias or anomalies that will be addressed through the calibration process, detailed in the following section [19].

**Table 5:** Sensor data metrics with units.

Sensor	Mean	Standard deviation	RMS	Peak-to-peak value	Skewness
AccX	0.1276 ${\mathrm{m/s}}^{2}$	0.0091 ${\mathrm{m/s}}^{2}$	0.1280 ${\mathrm{m/s}}^{2}$	0.2776 ${\mathrm{m/s}}^{2}$	−0.7342
AccY	0.0990 ${\mathrm{m/s}}^{2}$	0.0087 ${\mathrm{m/s}}^{2}$	0.0994 ${\mathrm{m/s}}^{2}$	0.1783 ${\mathrm{m/s}}^{2}$	−0.0767
AccZ	−9.7220 ${\mathrm{m/s}}^{2}$	0.0077 ${\mathrm{m/s}}^{2}$	9.7220 ${\mathrm{m/s}}^{2}$	0.1113 ${\mathrm{m/s}}^{2}$	−0.1538
GyroX	−0.0031 deg/s	0.0008 deg/s	0.0032 deg/s	0.0269 deg/s	−1.3964
GyroY	−0.0089 deg/s	0.0007 deg/s	0.0089 deg/s	0.0049 deg/s	−0.1080
GyroZ	−0.0037 deg/s	0.0009 deg/s	0.0038 deg/s	0.0232 deg/s	0.6859
MagX	−6.1295 μT	0.1683 μT	6.1318 μT	1.7720 μT	−0.0019
MagY	9.8572 μT	0.1695 μT	9.8587 μT	1.3610 μT	−0.0215
MagZ	9.5681 μT	0.3787 μT	9.5756 μT	2.5160 μT	0.2068
xNorth	0.06011E−4 m	6.7353 m	6.7352 m	63.3460 m	−4.0264
yEast	−0.00626E−4 m	3.2279 m	3.2278 m	14.6702 m	−0.7396
zDown	0.04391E−4 m	6.3106 m	6.3104 m	63.8002 m	1.8223
Barometer	0.0000 m	0.5931 m	0.5930 m	4.8359 m	−0.2609

### 7.2 Sensors calibration

#### 7.2.1 Magnetometer calibration

One common way to represent the Earth’s magnetic field is by recording the maximum measurements along each axis in every possible combination. Ideally, this representation should form a sphere centered at the origin, with maximum and minimum values of ±25 microteslas (μT) [20]. However, in practice, uncalibrated magnetometer data often shows ellipses with displaced centers and more pronounced axes. These deviations are attributed to Hard Iron Bias and Soft Iron Distortion [21].

**Fig. 14:**
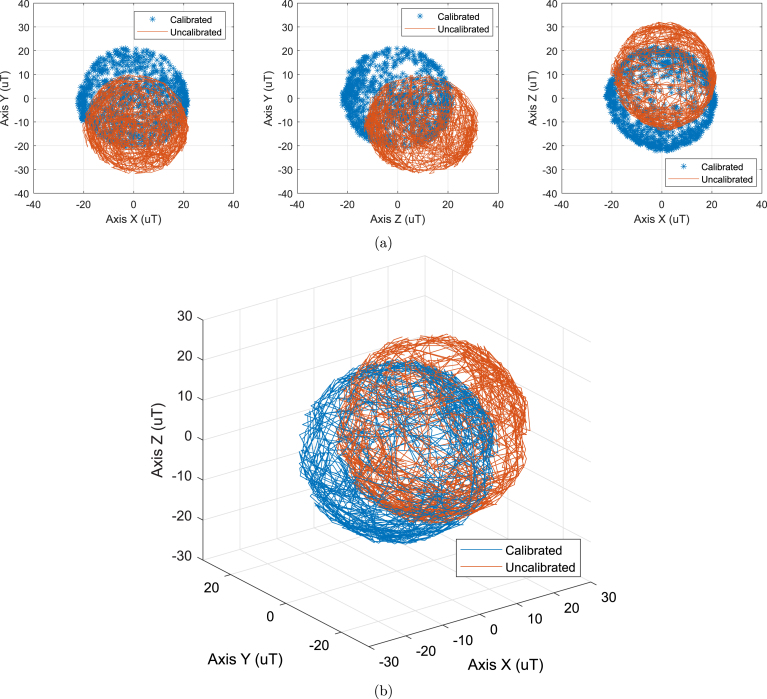
Representation of calibrated and uncalibrated magnetometer data. (a) Readings of the Earth’s magnetic field with calibrated and uncalibrated (HMC5883L, Honeywell, USA) sensor. (b) Comparison of calibrated and uncalibrated magnetometer readings.

Fig. 14 shows a comparison between the raw magnetometer data affected by hard and soft iron distortion in the surrounding area and the calibrated data. In this Figure, different views are presented to highlight the displacement that occurs experimentally in the environment where the authors conducted the tests. Fig. 14(a) shows the oval shape of the uncalibrated readings, while Fig. 14(b) represents the processed and unprocessed magnetometer data in 3D.

#### 7.2.2 Linear acceleration and angular velocity data

Before using commercial MEMS sensors for position and orientation estimation in an INS, proper calibration is essential. The operating instructions indicate that calibration of the accelerometer and gyroscope should be performed on a static surface without disturbances or movements. However, in the experimental environment, values named “offsets”, generated by small mechanical construction errors of the sensors, temperature, and their quality, are always obtained as was shown in Fig. 12 and Table 5 in the Skewness parameter [22].

**Fig. 15:**
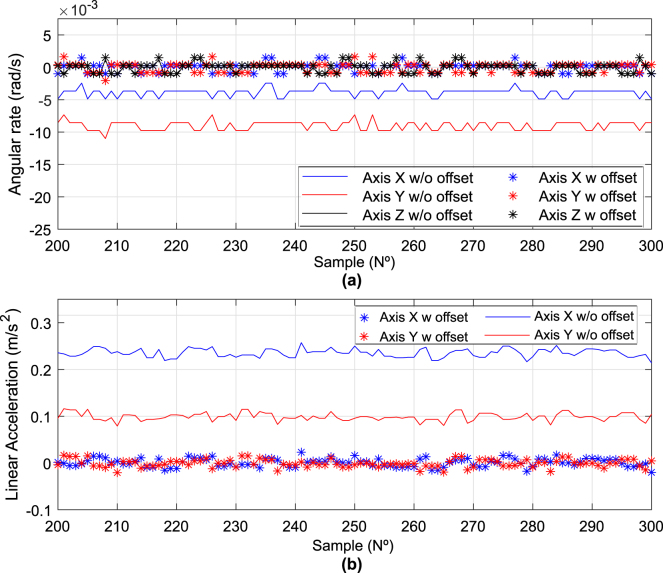
Comparison of raw IMU measurements with calibrated and filtered measurements. (a) Gyroscope data, (b) Accelerometer.

In this subsection, comparisons between raw data and offset-adjusted data are presented (Fig. 15). In the case of the gyroscope, readings of −10 milliradians per second are observed. These readings are adjusted to a smaller scale, approximating almost zero. It is important to note that although the signal quality has been improved, a small value still persists, which, when integrated over time, could cause drift error. However, this error can be covered by defining dead zones or implementing data quality improvement algorithms as proposed in previous works such as [23].

Regarding the accelerometer, the offset is much larger, and the Generalized Exponential Moving Average (EMA) filter was used to improve signal quality after offset compensation. In Fig. 15, comparisons between raw data and data adjusted with the EMA filter can be seen.

The EMA filter is widely used in discrete systems and is common for improving sensor signals with a lot of noise [24]. One of its main advantages is the computational cost required by this filter, compared to digital filters. The formula defined in (2) can be used to represent EMA filter.

(2) $$y_{\left[n\right]}=\alpha x_{\left[n\right]}+\left(1+\alpha \right)y_{\left[n-1\right]}$$

where $x_{\left[n\right]}$ is the current sample of the unfiltered signal, $y_{\left[n\right]}$ is the current sample of the filtered signal, $y_{\left[n-1\right]}$ is the previous sample of the filtered signal, and α is the weight assigned to the filter (0<α<1), where when α=1 the output will be equal to the input.

In the INS, within the TaskIMU task, this filter is used by calling a simple function in the C language. As observed, Eq. (2) uses basic operations such as sums and products with floating-point numbers and provides a significant improvement in signal quality, as in the case of the barometer (Fig. 18).

### 7.3 Application

**Fig. 16:**
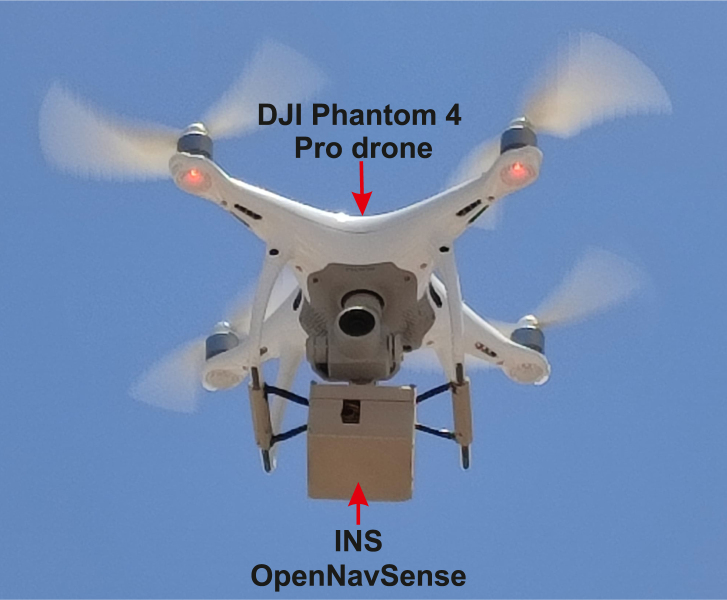
Physical implementations using the Phantom 4 pro drone in Arequipa, Peru.

For this study, flight maneuvers were conducted with the P4P drone in Arequipa, Peru. The P4P was chosen for its availability in the laboratory and its positioning accuracy, which offers a horizontal precision of ±0.3 m and a vertical precision of ±0.1 m [25], [26]. These levels of precision are adequate for using its recorded data as a reference for comparison with the platform’s data. During the flight tests, the drone carried the **INS OpenNavSense** platform as payload, as depicted in Fig. 16.

**Fig. 17:**
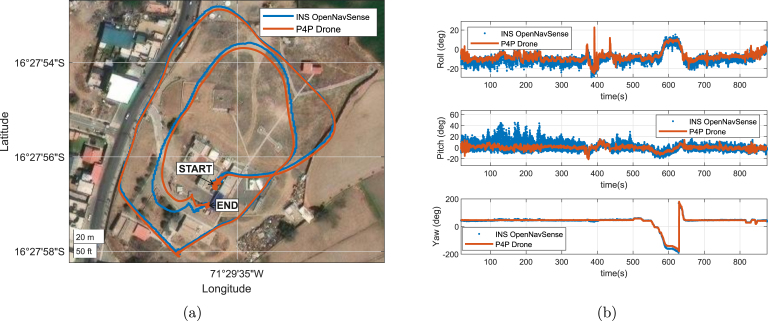
Position and orientation record of the OpenNavSense INS and drone during flight maneuvers. (a) Representation of the trajectory during tests, (b) Orientation during flight maneuvers of Yaw, Pitch, and Roll parameters.

Fig. 17 illustrates the drone’s trajectory over an uninhabited area (see Fig. 17(a)), marking the start and end points of the maneuvers. Fig. 17(b) shows the similarities between the inertial navigation system (INS) calculations of the drone and those of the open-source Mahony estimation algorithm. Notably, the **OpenNavSense** and P4P reference frames were aligned to ensure accurate comparison. High cross-correlation coefficients were observed in the Roll and Yaw angles, with values of 0.83 and 0.96, respectively, as summarized in Table 6. However, lower accuracy was noted for the Pitch angle, with a cross-correlation coefficient of approximately 0.3925. This discrepancy is attributed to significant noise in the measurements, particularly between 100 and 300 s in Fig. 17(b), where the drone’s flexible physical structure introduced oscillatory movement, amplifying accelerometer and gyroscope noise. This effect is most pronounced in the Pitch angle due to translational movements along the X-axis, causing oscillatory noise in pitch measurements.

**Fig. 18:**
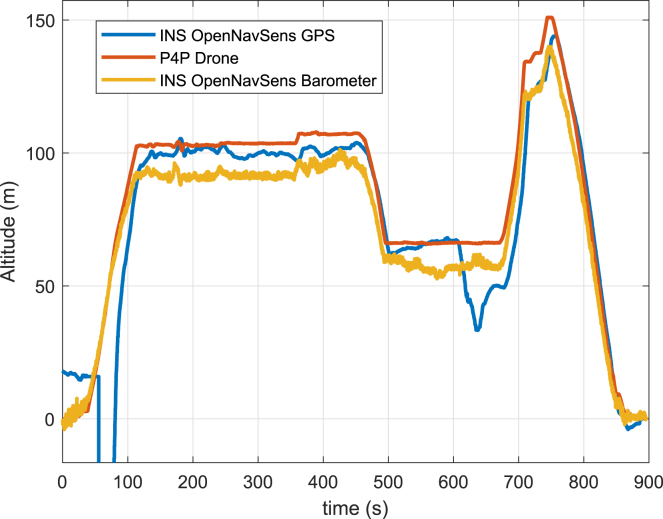
Comparison of the altitude recorded by the OpenNavSense INS using its barometer and GPS with the Altitude recorded by the Drone.

On the other hand, the altitude recorded by the GPS and the altitude calculated using barometer data with (1), filtered through an EMA filter with α=0.01, are analyzed alongside the P4P altitude as a reference. In Fig. 18, it can be observed that the barometer data demonstrates greater stability compared to the GPS module data. However, while GPS measurements offer better accuracy, notable disturbances are observed around 50 s and 650 s in Fig. 18 due to intermittent satellite connection loss. This analysis aligns with the parameters shown in Table 6, where, as in the previous section, the geodetic coordinates were transformed to NED coordinates relative to (−16.46561, −71.49306, 2.46795E3) using the MATLAB function geodetic2ned. In Table 6, a high cross-correlation coefficient (≥0.90) is found among Latitude, Longitude, GPS Altitude, Barometer Altitude, xNorth, and yNorth parameters.

Finally, Table 6 presents an important parameter called the Root Mean Squared Error (RMSE), which measures the error between the **OpenNavSense** and P4P data. The RMSE results indicate that several improvements to the navigation algorithm can be achieved using the proposed platform, which serves as the motivation for this study.

**Table 6:** Signal comparison metrics with RMSE and correlation coefficient.

Parameter	RMSE^a^	Cross-correlation coefficient^b^
Latitude (deg)	7.9653E−5	0.96707
Longitude (deg)	7.8011E−5	0.93454
GPS altitude (m)	10.7887	0.93384
Barometer altitude (m)	10.9386	0.99736
Roll (deg)	3.7513	0.83542
Pitch (deg)	7.6697	0.3925
Yaw (deg)	14.9917	0.96069
xNorth (m)	8.8182	0.96707
yNorth (m)	8.3336	0.93454

a RMSE of OpenNavSense with respect ton P4P.

b Cross correlation between OpenNavSense and P4P

### 7.4 Conclusion

This study presents the development of the *OpenNavSense* INS platform, demonstrating its potential as a cost-effective alternative capable of achieving functionality comparable to higher-end INS solutions. The platform was created using 3D-printed materials, low-cost Commercial Off-The-Shelf (COTS) electronics, and FreeRTOS as the operating system. This combination allows for the independent management of computational tasks, enabling students to easily test proposed INS algorithms using well-characterized open-source hardware. Therefore, the main goal of the paper is achieved with a manufacturing cost of approximately $50.

Additionally, a key difference from other similar devices, such as **AutoNAV** and **OpenIMU**, is that all necessary information for replication (both hardware and software) is available as open-source development code. This allows students with limited knowledge in programming, FreeRTOS, and electronics to focus primarily on implementing advanced INS filtering techniques, such as Kalman filtering and sensor fusion algorithms (e.g., [6]), to improve accuracy, without being concerned with the hardware.

However, despite the many advantages the proposed device offers, several key areas for improvement remain for future versions. First, as observed, the devices are relatively large due to the use of test boards and through-hole pin devices, which make replication easier for students. By integrating Surface-Mount Device (SMD) components, a more compact design could be achieved. Similarly, while the (LM7805, Texas Instruments, USA) regulator was used for convenience, it could be replaced with a switch-mode regulator to improve voltage conversion efficiency and extend battery life.

Another improvement would be to increase the operating frequency, as certain critical applications require higher frequencies. Enhancing the operating frequency would enable more accurate comparisons with tactical-grade systems such as the (VN-300, VectorNav Technologies, USA). Additionally, although calibration is rarely required, IMU and magnetometer calibration could be implemented directly within the (ESP32, Espressif Systems, China), eliminating the need for MATLAB and streamlining the workflow.

Finally, to overcome GPS signal loss and enhance position determination, GPS and barometer data could be combined with terrestrial radio measurements and odometers. This would improve reliability in GPS-denied conditions [10].

## CRediT authorship contribution statement

**Pablo Raul Yanyachi:** Writing – review & editing, Writing – original draft, Supervision, Resources, Project administration, Methodology, Investigation, Conceptualization. **Jorch Mendoza-Chok:** Writing – review & editing, Writing – original draft, Visualization, Validation, Software, Methodology, Investigation, Formal analysis, Conceptualization. **Brayan Espinoza-Garcia:** Writing – review & editing, Writing – original draft, Validation, Software, Methodology, Investigation. **Juan Carlos Cutipa Luque:** Writing – review & editing, Methodology, Formal analysis, Conceptualization. **Daniel Yanyachi Aco Cardenas:** Writing – review & editing, Resources, Methodology.

## Declaration of competing interest

The authors declare that they have no known competing financial interests or personal relationships that could have appeared to influence the work reported in this paper.

## Figures and Tables

**Table B.1:** Analysis of main microcontroller options for use in the OpenNavSense INS.

Criteria	ESP32	ESP8266	Arduino Uno	Raspberry Pi Zero
Processor	Dual-core 32-bit Xtensa LX6	32-bit Tensilica L106	8-bit AVR (ATmega328P)	ARM Cortex-A53 Single-core
Speed (MHz)	160–240 MHz	80 MHz	16 MHz	1 GHz
RAM	520 kB SRAM	160 kB	2 kB	512 MB
Connectivity	Wi-Fi, Bluetooth	Wi-Fi	No native (requires module)	Wi-Fi and Bluetooth (with adapter)
GPIO	34 pins	17 pins	14 pins	40 pins
Power	Low to medium	Low	Low	High
Ease of use	Medium	Medium	High	Medium
Approximate price	$5–$10	$2–$5	$10–$25	$10–$20
FreeRTOS capability	High, Native	Moderate	Low	Moderate to high

**Table B.2:** Analysis of IMU options for use in the OpenNavSense INS.

Criteria	MPU6050	MPU9250	LSM6DSOX	BMI160
Accelerometer range (g)	±2, ±4, ±8, ±16	±2, ±4, ±8, ±16	±2, ±4, ±8, ±16	±2, ±4, ±8, ±16

Gyroscope range (dps)	±250, ±500, ±1000, ±2000	±250, ±500, ±1000, ±2000	±125, ±250, ±500, ±1000, ±2000	±125, ±250, ±500, ±1000, ±2000

Magnetometer	No	Yes (AK8963)	No	No
Digital interface	I^2^C, SPI	I^2^C, SPI	I^2^C, SPI	I^2^C, SPI

Power consumption (mA)	3.9 mA (active)	3.1 mA (gyro + accel), 4.3 mA (with magnetometer)	0.9 mA (low-power), 0.65 mA (high-performance)	0.99 mA (low-power), 0.6 mA (full-performance)

RMS noise: Accelerometer	400μg/$\sqrt{\mathrm{Hz}}$	300μg/$\sqrt{\mathrm{Hz}}$	90μg/$\sqrt{\mathrm{Hz}}$	150μg/$\sqrt{\mathrm{Hz}}$

RMS noise: Gyroscope	0.005°/s/$\sqrt{\mathrm{Hz}}$	0.004°/s/$\sqrt{\mathrm{Hz}}$	0.003°/s/$\sqrt{\mathrm{Hz}}$	0.004°/s$\sqrt{\mathrm{Hz}}$

Typical applications	Basic motion tracking, robotics, wearable devices	Advanced motion tracking, IMUs, drones	Industrial IoT, fitness trackers, robotics	Wearable devices, mobile applications

Cost (USD)	Low (∼2–4)	Moderate (∼5–8)	Moderate (∼4–6)	Low (∼3–5)

**Table B.3:** Analysis of main GPS options for use in the OpenNavSense INS.

Criteria	BN880	NEO-6M	NEO-M8N	Ublox ZED-F9P
GNSS systems	GPS, GLONASS	GPS	GPS, GLONASS, Galileo, QZSS	GPS, GLONASS, Galileo, Beidou
Magnetometer	Yes (QMC5883)	No	No	No
Accuracy (CEP)	2.5 m	2.5 m	1.5 m	0.1 m (RTK)
Update rate (Hz)	10 Hz	1 Hz	10 Hz	10 Hz
Interface	UART, I^2^C	UART	UART, I^2^C	UART, I^2^C
Power consumption (mA)	28 mA	30 mA	23 mA	35 mA
Cold start (s)	30 s	27 s	26 s	25 s
Hot start (s)	1 s	1 s	1 s	1 s
Cost (USD)	Moderate (∼12–18)	Low (∼10–15)	Moderate (∼15–25)	High (∼200–300)

**Table B.4:** Analysis of barometer options for use in the OpenNavSense INS.

Criteria	BMP180	BMP280	BME280	LPS22HB
Pressure range (hPa)	300–1100	300–1100	300–1100	260–1260
Pressure accuracy (hPa)	±1.0	±0.12	±0.12	±0.1
Altitude accuracy (m)	1 m	0.5 m	0.5 m	0.3 m
Temperature sensor	Yes	Yes	Yes	Yes
Humidity sensor	No	No	Yes	No
Interface	I^2^C, SPI	I^2^C, SPI	I^2^C, SPI	I^2^C, SPI
Power consumption (μA)	12μA (typ.)	2.7μA (low-power mode)	2.7μA (low-power mode)	4μA (low-power mode)
Response time (ms)	7.5 ms	7.5 ms	7.5 ms	5 ms
Temperature accuracy (°C)	±2.0	±1.0	±1.0	±1.5
Cost (USD)	Low (∼3–5)	Low (∼3–6)	Moderate (∼4–8)	Moderate (∼4–8)

**Table C.5:** Definitions of statistical terms with units, formulas, and parameter descriptions.

Term	Definition	Units	Formula and Parameters
Mean	The average of all values in a dataset, calculated by summing all values and dividing by the total number of values. It represents the central tendency of the data.	Same as data units.	$\text{Mean}=\frac{1}{N}\sum_{i=1}^{N}x_{i}$

Standard deviation	A measure of the spread or dispersion of values around the mean in a dataset. A higher standard deviation indicates that values are more spread out from the mean.	Same as data units.	$\sigma =\sqrt{\frac{1}{N}\sum_{i=1}^{N}{\left(x_{i}-\text{Mean}\right)}^{2}}$

RMS (Root Mean Square)	The square root of the average of the squared values in a dataset. RMS is often used to calculate the effective value of a varying signal, such as voltage or current in electrical engineering.	Same as data units.	$\text{RMS}=\sqrt{\frac{1}{N}\sum_{i=1}^{N}x_{i}^{2}}$

Peak-to-peak value	The difference between the maximum and minimum values in a dataset. It represents the full range of the data.	Same as data units.	$\text{Peak-to-Peak}=x_{\text{max}}-x_{\text{min}}$

Population coefficient of skewness based on the third moment (skewness)	A measure of the asymmetry of the distribution of values in a dataset. Positive skewness indicates a distribution with a longer right tail, while negative skewness indicates a longer left tail.	Unitless	$\text{Skewness}=\frac{1}{N}\sum_{i=1}^{N}{\left(\frac{x_{i}-\text{Mean}}{\sigma }\right)}^{3}$

## Appendix A Mahony AHRS algorithm

The Mahony filter, described in [13], is an algorithm used to estimate attitude using measurements from gyroscopes, accelerometers, and magnetometers. The filter uses quaternions to represent the orientation of the rigid body. It employs a proportional-integral (PI) feedback filter to correct the gyroscope bias with the help of accelerometer and magnetometer measurements. In this subsection, a summary of this algorithm is shown [27].

An orientation quaternion $q={\left[q_{0},q_{1},q_{2},q_{3}\right]}^{T}$ is defined to represent the rotation of a device frame B relative to an immobile frame I. The vector $\omega ={\left[\omega_{x},\omega_{y},\omega_{z}\right]}^{T}$ denotes the angular rate measured by the gyroscope in (rad/s), which in practice corresponds to the angular rate of frame B relative to frame I, expressed in frame B.

According to [28], the derivative of the quaternion $\overset{˙}{q}$ is calculated using the ideal gyroscope data and the kinematic equation given by (A.1).

(A.1) $$\overset{˙}{q}=\frac{1}{2}q⊗\Omega$$

where ⊗ denotes quaternion multiplication and Ω is the quaternion form of the angular velocity as is shown in (A.2).

(A.2) $$\Omega ={\left[\begin{array}{cccc} 0 & \omega_{x} & \omega_{y} & \omega_{z} \end{array}\right]}^{T}$$

Replacing (A.2) in (A.1) and solving the quaternion multiplication, $\overset{˙}{q}$ is expressed as (A.3).

(A.3) $$\overset{˙}{q}=\frac{1}{2}\left[\begin{array}{c} -q_{1}\omega_{x}-q_{2}\omega_{y}-q_{3}\omega_{z}\\ q_{0}\omega_{x}+q_{2}\omega_{z}-q_{3}\omega_{y}\\ q_{0}\omega_{y}-q_{1}\omega_{z}+q_{3}\omega_{x}\\ q_{0}\omega_{z}+q_{1}\omega_{y}-q_{2}\omega_{x} \end{array}\right].$$

As a second step, the gravity and Earth’s Magnetic Field unitary vectors are used by the Mahony filter as reference vectors to reduce de offset.

Assuming that the unitary gravity vector of the Earth is always $g={\left[0,0,1\right]}^{T}$ in the frame I, the unitary estimated gravity vector $\overset{ˆ}{g}$ in the frame B can be calculated as (A.4). Where $\overset{ˆ}{q}$ is the estimated quaternion and ${\overset{ˆ}{q}}^{*}$ is its conjugate.

(A.4) $$\overset{ˆ}{g}=\overset{ˆ}{q}⊗g⊗{\overset{ˆ}{q}}^{*}$$

On the other hand, let $m_{\text{ref}}$ represent the Earth’s magnetic field unitary vector in the I frame. The estimated magnetic field unitary vector in the B frame is (A.5)

(A.5) $$\overset{ˆ}{m}=\overset{ˆ}{q}⊗m_{\text{ref}}⊗{\overset{ˆ}{q}}^{*}$$

The third step is to calculate the orientation error between the estimated unitary vectors ($\overset{ˆ}{g}$ and $\overset{ˆ}{m}$) and the measured unitary vectors (a from the accelerometer and m from the magnetometer).

The gravity error ($e_{\text{grav}}$) between $\overset{ˆ}{g}$ and a is denoted by (A.4).

(A.6) $$e_{\text{grav}}=a\times \overset{ˆ}{g}$$

where × denotes the cross product and the vector $e_{\text{grav}}$ represents the rotation needed to align $\overset{ˆ}{g}$ with the a vector. Similarly, the error between $\overset{ˆ}{m}$ and m is defined by (A.7).

(A.7) $$e_{\text{mag}}=m\times \overset{ˆ}{m}$$

The $e_{\text{mag}}$ vector represents the correction needed to align $\overset{ˆ}{m}$ with m. The total orientation error e is the sum of these errors as shown in (A.8).

(A.8) $$e=e_{\text{grav}}+e_{\text{mag}}$$

The Mahony filter uses a PI feedback control to correct the estimate orientation. The corrected angular rate $\omega_{\text{corrected}}$ is given by (A.9).

(A.9) $$\omega_{\text{corrected}}=\omega +K_{p}e+K_{i}\int e\;dt$$

where:

• $K_{p}$ is the proportional gain, controlling how quickly the filter corrects errors,
• $K_{i}$ is the integral gain, compensating for constant drift over time.

Finally, using the corrected angular rate $\omega_{\text{corrected}}$, the quaternion can be updated with (A.1).

## Appendix B Hardware selection tables

This Appendix presents the characteristics of the evaluated microcontroller and sensor options to be used in the OpenNavSense INS, shown in comparative Tables B.1, B.2, B.3, and B.4.

## Appendix C Statistic parameter definition

This Appendix in Table C.5 shows the statistical parameters used in Table 5 to characterize the INS OpenNavSens sensors. In Table C.5, $x_{i}$ represents the ith data point in the dataset, N is the total number of data points, $x_{\text{max}}$ is the maximum value in the dataset, and $x_{\text{min}}$ is the minimum value in the dataset [19], [29].
